# Health and well-being for all: an approach to accelerating progress to achieve the Sustainable Development Goals (SDGs) in countries in the WHO European Region

**DOI:** 10.1093/eurpub/ckaa026

**Published:** 2020-05-11

**Authors:** Bettina Menne, Emilia Aragon de Leon, Marleen Bekker, Nino Mirzikashvili, Stephen Morton, Amanda Shriwise, Göran Tomson, Pia Vracko, Christoph Wippel

**Affiliations:** c1 Health and Sustainable Development, WHO Regional Office for Europe, Copenhagen, Denmark; c2 Health and Society Group, Wageningen University and Research, Wageningen, the Netherlands; c3 WHO Country Office, Tbilisi, Georgia; c4 Healthy and Sustainable Settings Unit, University of Central Lancashire, Preston, UK; c5 Research Center on Inequality and Social Policy (SOCIUM), University of Bremen, Bremen, Germany; c6 Department of Sociology, University of Kansas, Lawrence, KS, USA; c7 President’s Office, Karolinska Institutet, Stockholm, Sweden; c8 Swedish Institute for Global Health Transformation (SIGHT), Royal Swedish Academy of Sciences, Stockholm, Sweden; c9 Health Systems Department, National Institute of Public Health, Ljubljana, Slovenia

## Abstract

**Background:**

Forty-three out of 53 of the WHO European Member States have set up political and institutional mechanisms to implement the United Nations (UN) 2030 Agenda for Sustainable Development. This includes governance and institutional mechanisms, engaging stakeholders, identifying targets and indicators, setting governmental and sectoral priorities for action and reporting progress regularly. Still, growing evidence suggests that there is room for advancing implementation of some of the Sustainable Development Goals (SDGs) and targets at a higher pace in the WHO European Region. This article proposes the E4A approach to support WHO European Member States in their efforts to achieve the health-related SDG targets.

**Methods:**

The E4A approach was developed through a 2-year, multi-stage process, starting with the endorsement of the SDG Roadmap by all WHO European Member States in 2017. This approach resulted from a mix of qualitative methods: a semi-structured desk review of existing committal documents and tools; in-country policy dialogs, interviews and reports; joint UN missions and discussion among multi-lateral organizations; consultation with an advisory group of academics and health policy experts across countries.

**Results:**

The E—engage—functions as the driver and pace-maker; the 4 As—assess, align, accelerate and account—serve as building blocks composed of policies, processes, activities and interventions operating in continuous and synchronized action. Each of the building blocks is an essential part of the approach that can be applied across geographic and institutional levels.

**Conclusion:**

While the E4A approach is being finalized, this article aims to generate debate and input to further refine and test this approach from a public health and user perspective.

## Introduction

**Figure 1:**
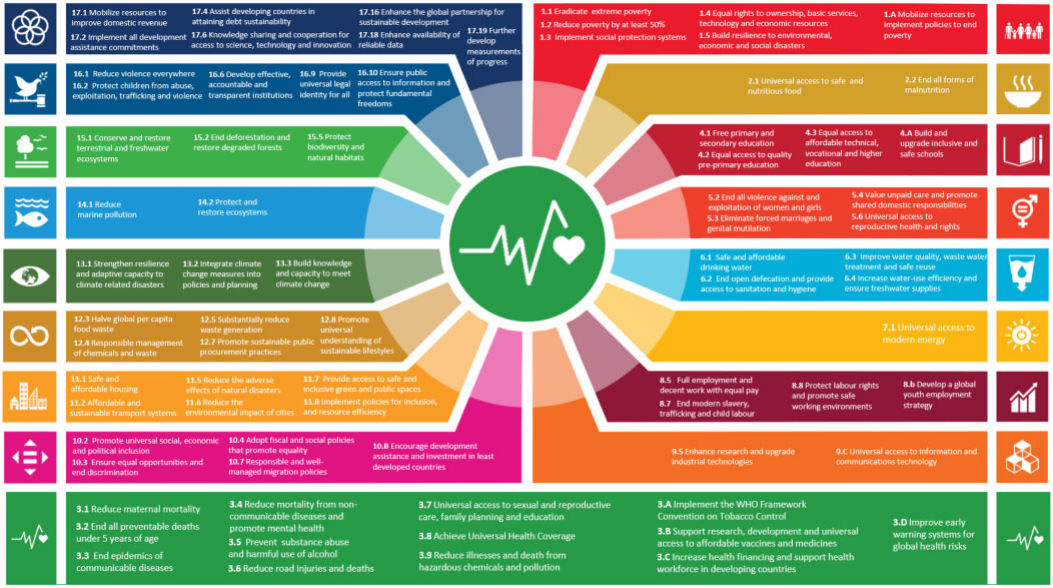
Health-related SDG targets (World Health Organization Regional Office for Europe, 2019)

Almost 5 years have passed since the 2030 Agenda for Sustainable Development and its Sustainable Development Goals (SDGs) were adopted by all 193 Member States of the United Nations (UN). The 2030 Agenda presents a universal call to action to end poverty, protect the planet and ensure that all people enjoy peace and prosperity by 2030.^1^ The 2030 Agenda consists of 17 SDGs and 169 targets that are universal, interconnected and indivisible. Health is both an enabler, and a major outcome, of sustainable development. SDG 3 is dedicated to health and well-being for all at all ages, and it has 13 targets. Achieving SDG 3 will only be possible if action in other sectors and settings is also advancing, such as efforts to end poverty (SDG 1), zero hunger (SDG 2), quality education (SDG 4), gender equality (SDG 5), clean water and sanitation (SDG 6), affordable and clean energy (SDG 7), decent work and equitable economic growth (SDG 8), industry, innovation and infrastructure (SDG 9), reduction of inequalities (SDG 10), sustainable cities and communities (SDG 11), sustainable production and consumption (SDG 12), climate action (SDG 13), life below water (SDG 14), life on land (SDG 15), peace (SDG 16) and governance (SDG 17). There are numerous health-related targets across the other 16 SDGs (figure 1).^2^

Over the last decades, remarkable gains have been made in increasing overall and healthy life expectancy, reducing maternal and child mortality, increasing the capacity for early warning, risk reduction and management of national and global health risks and in reducing the burden of communicable and non-communicable diseases (NCDs) in the WHO European Region.^3^ Implementation of the 2030 Agenda is advancing in all WHO European Member States, but current projections indicate that no country is fully on track to achieve the health-related targets and SDGs and that there is room for further strengthening and advancing implementation at a higher pace.^3^^,^^4^ Some health targets in SDG 3 and other health-related targets will be achieved only if action is accelerated across the whole of government and society. This includes halving the number of global deaths and injuries from road traffic accidents, reducing multi-drug-resistant tuberculosis and new HIV infections, scaling up immunization rates, tackling risk factors like obesity, alcohol, tobacco use and air pollution, addressing mental health disorders and reducing interpersonal violence.^3^

Universal health coverage, or ensuring that everyone can use the quality health services they need without experiencing financial hardship, is essential. Major components of universal health coverage include health service coverage, financial protection, primary health care, a well-trained and distributed health workforce and access to affordable, effective and quality medicines, and these are of great concern to European Member States. Antimicrobial resistance, environmental pollution and climate change, gaps in social protection and inequities in living conditions, economic disparities, lack of political will and institutional capabilities and fragmentation are putting stress on people and on achieving health and well-being for all at all ages. On top of this, ‘Health inequities and the distribution of health risks and conditions remain unacceptably uneven’, and the rate of improvement ‘to reduce avoidable gaps in health is slower than anticipated and below what is possible given existing knowledge and commitments’.^5–7^

Recognizing its unprecedented ambition, scale, universality and opportunities, all WHO European Member States endorsed the Roadmap to implement the 2030 Agenda in 2017.^8^^,^^9^ The SDG Roadmap aims to strengthen the capacities of Member States to achieve better, more equitable, sustainable health and well-being for all at all ages in the WHO European Region. The SDG Roadmap reflects that the 17 SDGs and their 169 targets imply vertical integration (global, national, community and individual) and horizontal integration (across all SDGs and the economic, social and environmental domains of sustainable development). The SDGs provide opportunities for public health stakeholders to act early and upstream, to create cultures of well-being, to engage in planetary health and to strengthen disease prevention, health protection and health promotion.^10^

In 2018, experts and representatives from European Member States, WHO and other UN agencies noted that it is challenging to mainstream health and to operationalize the SDGs due to their cross-cutting nature. To do so requires new approaches to vertical and horizontal integration. They recommended existing knowledge and resources on SDG implementation be integrated into a single approach that would support Member States in delivering the 2030 Agenda.^11^ In parallel, a process at the global level was initiated by 12 multi-lateral health, development and humanitarian agencies to develop the Global Action Plan for Healthy Lives and Well-being for All (GAP),^12^ to strengthen collaboration among multi-lateral organizations and to accelerate country progress on the health-related SDG targets.

In sum, implementing the SDGs requires a new collective approach, not confined to the health sector but with engagement of actors and sectors who do not traditionally share mutual objectives. This article presents the E4A approach—engage, assess, align, accelerate and account. The E4A approach aims to support countries in the WHO European Region to achieve the health-related SDG targets. The E4A approach is a synchronous approach to policy development and implementation designed to support policy makers, public health institutions and professionals in accelerating progress in line with the 2030 Agenda. As this approach is further refined and finalized, the article aims to generate discussion between public health stakeholders and academics across disciplines and to encourage pilots of this approach in countries.

## Methods

**Figure 2:**
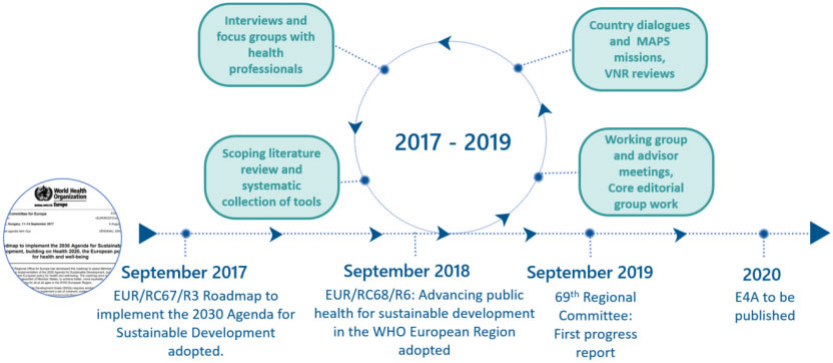
Timeline of the development of the E4A approach (World Health Organization Regional Office for Europe, 2019)

A 2-year, multi-stage process with a mix of qualitative methods was carried out. It started with the endorsement at the 67th and 68th Regional Committees for Europe of the SDG Roadmap (2017)^8^^,^^9^ and of the action plan to advance public health for sustainable development (2018) (figure 2).^10^^,^^13^

To understand the tools and instruments available to support European Member States in implementing the SDGs, four desk reviews and assessments were conducted. First, a desk review of documents from the World Health Assembly, WHO Regional Committee for Europe and the UN identified 196 committal documents (meaning endorsed by WHO or UN Member States) between 1990 and 2017.^14^ Second, a semi-structured desk review of existing tools on the WHO’s digital library, the UN Sustainable Development Group’s SDG Acceleration Toolkit and the UN Development Programme’s library was conducted, which returned ∼800 tools. Two sets of criteria were then established for inclusion: (i) tools that were published in the last 5 years and (ii) tools that were deemed practical, useful for decision-making across different areas of health, supportive of actions and interventions to achieve SDGs and targets and that had the potential to accelerate implementation across the SDGs and targets. This resulted in a list of 400 tools, categorized by four WHO experts into (i) SDGs and target(s), (ii) the SDG Roadmap’s strategic directions and enablers and (iii) stages of the policy cycle. Classification conflicts were resolved through consensus judgments. Third, in preparation of the first progress report on the implementation of the SDG Roadmap in 2019,^3^ two additional reviews were undertaken on (i) SDG governance arrangements and health content as described in 43 Voluntary National Reviews (VNRs) conducted by WHO European Member States and (ii) the extent to which the actions taken in 20 countries, as described in their VNRs, align with the Roadmap’s recommendations.^15^ Fourth, an analysis on the status of each of the SDG 3 targets and on several other health-related SDG targets, their interconnectivity with other SDGs, available policy instruments, indicators and resources was conducted.^16^^,^^17^

Work in countries across the European Region was an integral part of this 2-year process. Eight UN mainstreaming, acceleration and policy coherence (or UN MAPS) missions in European Member States informed this approach.^18^ These missions included multi-sectoral assessment of the countries’ implementation of the SDGs, an analysis of national policies against the SDG targets (known as Rapid Integration Assessment)^19^ and structured, multi-sectoral policy dialogues and guided interviews with line ministries, civil society and parliaments. During this process, discussion occurred with around 250 multi-sectoral stakeholders and key actors on how to best mainstream health and well-being. In addition to the UN MAPS missions, expert advice and work in many additional countries and across UN agencies informed WHO planning frameworks and vision documents to support evidence-informed decision-making on health-related SDG implementation. Guidance notes developed by international partners (e.g. the Organisation for Economic Co-operation and Development, among others) also contributed to the development of this approach.

Together, these activities led to the development of a preliminary version of a new approach to policy development and implementation that was reviewed in June 2019 at a multi-disciplinary expert meeting of representatives from governments, academia, civil society organizations, WHO and other UN agencies.^20^ The aim was to test and improve the user experience of this policy approach as an implementation tool.

To ground, connect and further refine this model in line with recent academic literature, a semi-structured literature review was also performed searching for articles via Google Scholar based on the following key terms: (i) health and sustainable development and (ii) health policy implementation. This search returned 56 peer reviewed articles published since 2017 and a few additional articles on policy and governance.

## Results

The analysis of the 43 VNRs—the official UN reporting mechanism on progress in achieving the SDGs in countries—revealed new governance mechanisms put in place, with most countries reporting a change in the responsibility of oversight, monitoring and/or coordination of the sustainable development activities from the Ministry for Foreign Affairs or Ministry for Environment to the Prime Minister’s office or another central office or body. There is also evidence of engagement by civil society, the private sector and/or local authorities in several countries, illustrating a pluralistic approach to implementation.^3^

While inter-ministerial and/or intersectoral oversight, monitoring and/or coordination mechanisms and processes have been established, implementation of the SDGs continues to be delivered largely within line ministries, with only a few countries reporting truly intersectoral actions. The framing of health policy within the SDGs in the analyzed high-income countries has shifted noticeably from being primarily an issue of external aid for the global development agenda to becoming an issue of domestic policy. With some notable exceptions, health appears to still be treated as merely one of the 17 Goals. Implementation of the SDGs is building on existing governance structures, with the Health Ministry and its institutions having the main responsibility in taking action to achieve SDG 3. There is a risk that weaknesses of current health governance structures and change resistant behavior may be perpetuated, which may hamper needed transformation.^7^

Multi-sectoral policy dialogs and interactive expert meetings highlighted the need to balance governance with innovation and to ensure the integration of the three domains of sustainable development into health governance.

Recent literature highlights how SDG implementation could deliver health gains in areas such as child health,^21^ mental health,^22^ prevention of NCDs^23^ and from tackling climate change.^24^ Additional studies have looked at health systems,^25^ primary health care,^26^ development assistance for health^27^ and transnational interactions as political determinants, with a potential to address multiple SDGs and proving a nexus to complex inter-relationships.^7^

The analysis of the tools and instruments available to support WHO European Member States to implement the SDGs highlighted that there is a vast amount of guidance for both Member States and health professionals, focusing on specific health-related targets and priorities, but that there is a gap in an overarching approach, where they are logically pulled together.

To address this gap, we developed an approach that integrates societal transformative change with policy implementation at the systems level. The approach builds on the cyclical four-step PDCA (plan, do, check, act) model, which is used in management for change and quality improvement at the process level and complements it in the governance of societal transformative change.^28^ It also shares parallels with the PDIA (problem-driven iterative adaptation) model, which focuses on locally identifying and prioritizing implementation problems in development policy.^29^

**Figure 3:**
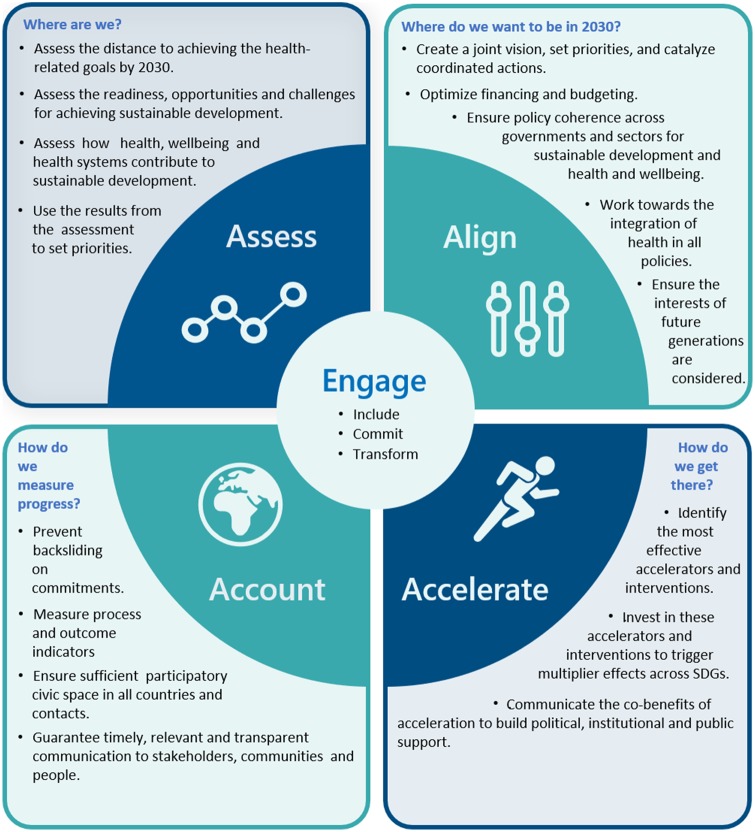
The E4A approach to achieve the SDGs (World Health Organization Regional Office for Europe, 2019)

The E4A approach consists of five building blocks: engage, assess, align, accelerate and account. Engage is the pace-maker that drives action; the 4As are building blocks and their elements can be used in a continuous and synchronized way (figure 3). While all of these parts should be considered during implementation, some may be stressed or relied on more than others, depending on the needs and priorities of each country. The E4A approach can include elements of experimentation (assessing new ideas and joint initiatives),^30^ collaboration,^31^ reflexive deliberation (aligning global norms and public and societal values) and adaptation.

ENGAGE refers to the systematic and meaningful engagement with health-related stakeholders across all sectors and levels. Institutional rigidity as well as systemic and capacity-related challenges can slow or inhibit meaningful engagement. Depending on the power and interests of health stakeholders and other key actors in relation to health and well-being, engagements can be strategically planned^32^ to raise awareness, empower, advocate and promote close engagement. Stakeholder engagement and communication plans are useful instruments to support these endeavors.

ASSESS refers to the process of understanding the distance to achieving the health-related SDG targets, as well as the context, opportunities and challenges to achieving them. It is suggested that assessment includes an SDG diagnostic (measuring current trends and the distance to achieving the health-related SDG targets, minding their inter-linkages), an SDG readiness analysis (understanding the current alignment of existing policies and measures with the SDGs as well as identifying gaps and bottlenecks),^19^ examination of the interactions between the SDGs and their targets^33^ and an understanding of health governance in the context of the 2030 Agenda. Strong assessment of the health status within a country is more than thick description; it provides a detailed and nuanced understanding of the context in which engagement takes place in a way that facilitates planning and that identifies windows of opportunity for action at all levels of governance.^34^

ALIGN refers to the harmonization of policies and processes within and between sectors and levels of governance related to the achievement of the health-related SDG targets. Alignment starts with the existence or creation of a joint vision for health and well-being that maximizes co-benefits across the SDGs, minimizes the sustainability tradeoffs generated by activities within the health sector and harmonizes financial, legal and regulatory mechanisms. Continuous and sustained efforts at alignment, often supported by policy dialogs within and beyond the health sector, promote policy coherence in implementation^35^ and require institutional capacity and evidence-based knowledge. Important aspects, like health equity, gender and human rights, accountability and the outlook of young and future generations are important elements of alignment.^6^ Alignment has been recognized as critical to advancing health through a number of global legally binding instruments and UN resolutions or common positions, including for example the global implementation of the Framework Convention on Tobacco Control,^36^ the International Health Regulations and agreements on both communicable diseases and NCDs, most recently through the UN Common Position on Ending HIV, TB and Viral Hepatitis.^37^

**Figure 4:**
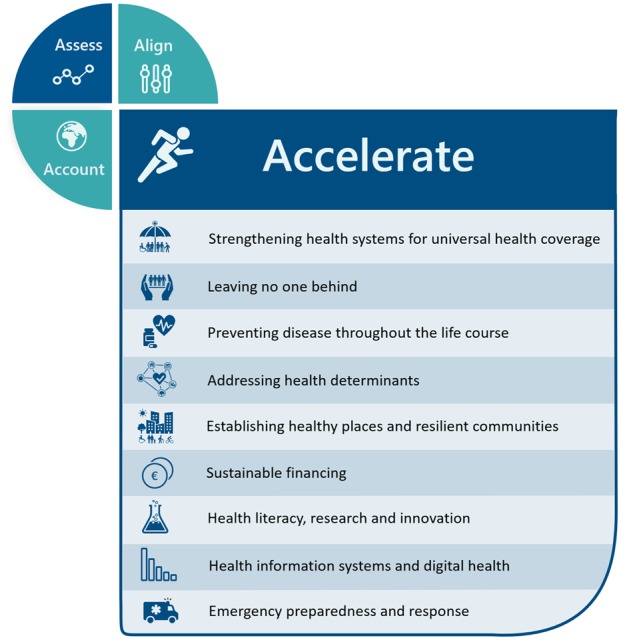
The proposed accelerators, partially derived from the SDG Roadmap (World Health Organization Regional Office for Europe, 2019)

ACCELERATE consists of catalytic elements and selected policy and/or program areas that can trigger positive multiplier effects across the SDGs and targets and that can increase the pace and support uptake of innovation in reaching the health-related SDG targets at all levels.^20^ Acceleration is context specific, highlighting the importance of tailoring SDG implementation to a system’s characteristics and to the institutional conditions and specific health needs of national and subnational environments. To identify country relevant accelerators, it is suggested that special attention is given to health equity, human rights and joint action in partnership. Drawing from Health 2020, GAP and discussions with European Member States, nine accelerator areas have been proposed, based on the strategic directions and enablers of the SDG Roadmap (figure 4).

ACCOUNT recognizes shared responsibility for implementing the 2030 Agenda and achieving its health-related SDG targets. Accountability helps to prevent backsliding on commitments, to de-politicize health information and statistics and to ensure sufficient participatory civic space in all countries. In the 2030 Agenda, governments are answerable for strategies and outcomes to achieve the SDGs. This includes legal obligations, financial accountability and tracking government commitments and pledges to improving health and sustainable development, human rights, gender equality and leaving no one behind. The media are critical for ensuring that both governmental and nongovernmental actors fulfill their political commitments and are held accountable by the public. Transparent and timely communication of information relevant to health and well-being facilitates the dissemination of accurate measures of health status and progress to ensure accountability.

## Conclusions

Discussion within countries across the European Region suggests that the 2030 Agenda is distinguished, in part, by the way in which its implementation is shaped by global norms as well as public and societal values and political priorities. It remains unclear how this new implementation paradigm is being put into practice.^38^ This presents challenges and opportunities to encourage the transformative change needed to achieve the SDGs and to strengthen value-based governance.^39^ How countries approach policy implementation has important governance implications for transparency and accountability and rights-based approaches. Benefits of enhancing value-based governance at national, sub-national and local levels include increased visibility, readiness and operational capacity and enforcement capabilities.^40^

We recognize the limitations in trying to find a pragmatic approach to answering a range of complex questions, including for example the role of primary health care in tackling wider health determinants (including environmental and economic determinants);^26^ the need for health in all policies; the role of global governance, commitments to global solidarity and shared responsibility; the growing recognition that global economic and political systems require a healthy planet and healthy people.^7^

In order to meet these challenges, we have proposed the E4A approach as a way of drawing lessons from governance practice across different countries and systems. The E4A approach is supported by work on value-based governance (align),^40^^,^^41^ policy implementation from a governance perspective (accelerate)^42^ and emerging narrative monitoring, evaluation and accountability through policy feedback and feedforward (assess and account). Incorporating health in all SDGs requires new ways of working, which can be challenging and may result in change resistant behavior. To overcome barriers and patches of resistance, the E4A approach might need to further incorporate five public value characteristics that can influence public acceptance in different policy stages: visibility, capacity to operationalize (e.g. prevention), capacity to enforce, duration term of achievement and level of contestation.^40^

There is an urgent need to reinforce health governance across the economic, environmental and social domains of sustainable development. Developing and strengthening coherent national public health strategies, policies and measures and ensuring that they are aligned with national development objectives and vice versa^7^ is one way to do this. The E4A approach might be useful in these processes through its engagement and alignment elements.

In line with the SDGs, the E4A approach provides a value-based and practical framework for public health action. It presents opportunities to broaden partnerships, strengthen cooperation, promote investments in public health policies and interventions, strengthen institutional capacities for the generation of evidence and promote communication to empower people to make healthy decisions for themselves and their families. Through its engagement and assessment components, the E4A approach facilitates a better understanding of the benefits of acting early and upstream, highlighting areas where policy coherence for sustainable development is missing; through its alignment and acceleration components, it promotes the creation of cultures of health and well-being. It will also contribute to the creation of a new, fit for purpose public health workforce.

The method of development of this approach has a number of limitations. While it is a promising and workable tool that is evidence-informed and based on multi-sectoral expert opinion, it has not yet been tested in a country context. It is at a proof of concept stage, and more evidence on its impact and exploration of its validity in countries across the WHO European Region is needed. Can it be used equally from Tajikistan to Sweden? We thus welcome comments on this approach and volunteers from Member States and public health institutions to test it, either on their own or as part of wider collaboration (e.g. national or subnational) on SDG implementation.
